# Community-Academic Partnership to Improve Nutrition and Blood Pressure in Seniors: Outcomes & Impact of COVID-19

**DOI:** 10.1093/geroni/igab046.2323

**Published:** 2021-12-17

**Authors:** Dozene Guishard, Rhonda Kost, Jonathan Tobin, Kimberly Vasquez

**Affiliations:** 1 Carter Burden Network, New York, New York, United States; 2 Rockefeller University, New York, New York, United States; 3 Clinical Directors Network, New York, New York, United States; 4 The Rockefeller University, New York, New York, United States

## Abstract

The Dietary Approaches to Stop Hypertension (“DASH diet”) has been proven in research settings to lower blood pressure, but its implementation is untested among seniors in congregate meals settings. We report the planning, implementation, impact of COVID-19, and results of an Administration of Community Living-funded study to test whether two evidence-based interventions - DASH-alignment of congregate meals, and home blood pressure self-monitoring, can lower systolic blood pressure and increase blood pressure control among community-dwelling seniors.. Congregate meal menus were aligned with the DASH eating plan, through collaboration of Bionutrition professionals on the research team, CBN food services leadership, and the NYC Department for the Aging. Seniors provided feedback on the DASH-modified meal options. The intervention began on October 15, 2019 (Site 1) and February 3, 2020 (Site 2). The study was interrupted by the COVID-19 pandemic in March 2020, when congregate meals ceased, and when approximately 75% of primary outcome data were collected. Modified implementation permitted completion of modified study outcomes. Preliminary analyses suggest that some participants were able to lower their blood pressure in this program. The DASH diet implemented in the congregate meal setting, along with programs to support BP self-efficacy through modification of existing programs, may be a valuable and scalable model to reduce cardiovascular risk among community-living seniors.

